# Angle-Dependent Dip Coating Strategy for Silver Nanostructured Surface Fabrication with Enhanced Fluorescence and Surface-Enhanced Raman Scattering Properties

**DOI:** 10.3390/bios16050292

**Published:** 2026-05-16

**Authors:** Longchao Qi, Kaibo Guo, Xianlong Ning, Yiming Huang, Xun Lu

**Affiliations:** Department of Mechanical Engineering, Yanbian University, Yanji 133002, China; 2023010094@ybu.edu.cn (L.Q.); 2023010088@ybu.edu.cn (K.G.); 2023010093@ybu.edu.cn (X.N.); 2024010102@ybu.edu.cn (Y.H.)

**Keywords:** angle-dependent dip coating, nanoparticle self-assembly, LSPR, MEF, SERS

## Abstract

Noble metal nanostructures based on localized surface plasmon resonance (LSPR) can induce metal-enhanced fluorescence (MEF) and surface-enhanced Raman scattering (SERS), significantly improving trace detection sensitivity for biomedical and chemical analysis. While self-assembly of noble metal nanoparticles offers simplicity and low equipment dependence, achieving large-area, uniform, and controllable nanostructures remains challenging. In this study, angle-dependent dip coating (ADDC) technology was employed to achieve efficient, controllable self-assembly of silver nanoparticles (AgNPs) on glass slides, establishing a fabrication process for MEF/SERS dual-functional substrates. A stable AgNPs-anhydrous ethanol suspension was prepared and extracted from an inclined substrate reservoir using a microfluidic syringe pump, enabling large-area uniform nanostructure assembly. Systematic investigation revealed that substrate inclination angle provides better morphology and fluorescence enhancement control than withdrawal flow rate. The silver nanostructured surface fabricated under a withdrawal flow rate of 16 mL/h and a substrate inclination angle of 30° exhibited a Cy3 detection limit as low as $10^{-1}$ nM, with an enhancement factor ranging from 19.14 to 28.66, as well as an R6G SERS detection limit of $10^{-10}$ M with an enhancement factor of 4.07 × $10^{8}$. This study confirms that ADDC technology enables simple, efficient, large-area uniform AgNPs self-assembly for superior dual-function enhancement substrates, offering a cost-effective and efficient strategy for highly sensitive trace detection.

## 1. Introduction

Localized surface plasmon resonance (LSPR) refers to the optical phenomenon in which the free electrons of noble metal nanoparticles, such as gold and silver, undergo collective oscillations at the interface with the surrounding dielectric under incident electromagnetic excitation [1,2]. Based on the LSPR effect, metal-enhanced fluorescence (MEF) [3,4] and surface-enhanced Raman scattering (SERS) [5,6] substrates have been extensively developed, enabling significantly improved sensitivity for trace detection and demonstrating broad application prospects in biomedicine [7,8,9,10] and environmental monitoring [11,12,13,14,15,16].

Among the nanostructure fabrication strategies for MEF and SERS substrates, top-down nanofabrication techniques are the most representative approaches [17,18], including electron beam lithography [19], nanoimprint lithography [20,21], as well as noble metal thin-film deposition techniques integrated with these methods [22,23]. These approaches offer significant advantages in the precise control of nanostructure size, morphology, and periodicity, thereby enabling excellent signal enhancement performance and high signal uniformity [24,25]. However, they generally rely on expensive and sophisticated equipment, involve complex fabrication processes, and suffer from low throughput and high cost, which limit their scalability to some extent.

In contrast, self-assembly of noble metal nanoparticles has been widely employed for constructing nanostructures due to its cost-effectiveness, operational simplicity, and reduced reliance on expensive equipment. Such strategies primarily rely on interparticle interactions, including van der Waals forces, capillary forces, electrostatic interactions, hydrogen bonding, and hydrophobic effects, to regulate nanoparticle distribution and aggregation at interfaces or on substrate surfaces, thereby enabling nanostructure formation [26,27,28,29]. Current approaches include drop casting, Langmuir-Blodgett (LB) film transfer, liquid–liquid interfacial self-assembly, and dip coating and its variants such as angle-dependent dip coating (ADDC). Drop casting relies on droplet spreading and solvent evaporation on solid substrates, where capillary flow and particle migration drive nanoparticle deposition within seconds to minutes, offering operational simplicity and rapid processing. Therefore, drop casting is well suited for small-batch fabrication at the laboratory scale. For example, Sinha et al. deposited Ag nanoparticle aggregates onto PDMS-coated glass substrates and suppressed the coffee ring effect by controlling relative humidity, enabling facile fabrication of hydrophobic SERS substrates [30]. Wang et al. further demonstrated sensitive detection of inorganic ions and organic molecules by sequentially depositing silver nanoparticle (AgNP)-containing droplets onto hydrophobic silicon substrates [31]. However, evaporation-driven deposition is prone to coffee ring effects and random aggregation, resulting in limited uniformity and reproducibility. In comparison, LB film transfer improves structural uniformity by exploiting interfacial self-assembly at the air-liquid interface, where controlled compression enables the formation of dense and ordered monolayers or multilayers that can be transferred onto solid substrates [32,33]. For instance, Tim et al. assembled Au nanoparticles onto functionalized substrates via LB transfer and subsequent electroless gold plating to fabricate quasi-two-dimensional nanolattice SERS substrates for biomolecular detection [34]. Nevertheless, this method involves multiple steps, including surface modification, interfacial spreading, slow compression, and vertical transfer, leading to stringent processing conditions and limited scalability. Furthermore, liquid–liquid interfacial self-assembly further simplifies the interfacial construction and operational procedures, thereby improving the fabrication efficiency of nanoparticle self-assembly. This method utilizes the difference in surface tension at the interface between two immiscible phases to drive nanoparticles to accumulate at the interface and form densely packed structures, enabling rapid assembly within several minutes to tens of minutes [35]. Tan et al. fabricated Ag nanostructure arrays on sandpaper-molded PDMS substrates using this approach, achieving effective SERS performance for detecting organic dyes in environmental samples [36]. However, transferring the assembled structures from the liquid–liquid interface onto solid substrates requires precise control; otherwise, local inhomogeneity or structural disruption may occur, affecting reproducibility and fabrication efficiency. Therefore, developing self-assembly strategies that combine simplicity, scalability, and structural controllability is of great importance for high-performance nanostructured substrates. Among these approaches, dip coating is an efficient and straightforward method capable of producing large-area and uniform nanostructures using simple equipment without complex interfacial regulation or multistep pretreatment [37,38]. By adjusting parameters such as coating speed, interparticle spacing can also be effectively tuned. In a typical dip coating process, a substrate is withdrawn from a nanoparticle suspension at a constant speed, forming a uniform liquid film on its surface. During solvent evaporation, nanoparticles migrate, accumulate, and self-organize, ultimately forming a uniform nanostructure [39,40]. However, conventional dip coating provides limited control over film thickness and assembly behavior, and improvements in structural uniformity and fabrication efficiency are still needed. ADDC introduces substrate inclination angle as an additional parameter, enabling enhanced control over liquid film thickness and nanoparticle spacing, and improving structural uniformity, reproducibility, and fabrication efficiency [41,42,43]. Although significant progress has been made in theoretical modeling and optical coating fabrication using this method [44], its application to plasmonic enhancement substrates such as MEF and SERS, including controllable fabrication, parameter optimization, and performance evaluation, remains insufficiently explored.

Based on the above considerations, this study employs ADDC to achieve the efficient, large-area, and uniform self-assembly of AgNPs on substrate surfaces, aiming to construct dual-functional silver nanostructured surfaces with both MEF and SERS capabilities. First, an AgNP-ethanol suspension with good dispersion and stability was prepared to meet the requirements of the ADDC process. Subsequently, the suspension was continuously withdrawn at a controlled flow rate using a microfluidic syringe pump from a reservoir containing a substrate tilted at a defined inclination angle. The gradual recession of the liquid level drove the enrichment and self-assembly of AgNPs onto the glass substrate, forming large-area, uniformly distributed silver nanostructured surfaces with controllable interparticle spacing. During this process, the effects of key parameters, including substrate inclination angle and withdrawal flow rate, on surface morphology and fluorescence enhancement performance were systematically investigated, and the optimal fabrication conditions were determined. Finally, Cy3 and Rhodamine 6G (R6G) were employed as model probe molecules to evaluate the fluorescence and Raman enhancement performance, as well as the practical sensing capability of the substrates. The results demonstrate that, compared with conventional dip coating primarily governed by withdrawal rate, the introduction of substrate inclination angle significantly enhances the controllability of nanostructure morphology and fluorescence performance. The AgNP substrates fabricated via ADDC exhibit pronounced enhancement in both fluorescence and Raman signals for Cy3 and R6G, together with excellent detection performance. This work systematically evaluates the effectiveness and potential of ADDC for plasmonic substrate fabrication, providing a reliable experimental basis for its application in MEF and SERS platforms.

## 2. Materials and Methods

### 2.1. Main Reagents and Instruments

Silver nanoparticles (AgNPs, 20 nm, 99.99%) were purchased from Guangzhou Metal Metallurgy Co., Ltd. (Guangzhou, China). Absolute ethanol (≥99.8%) was obtained from Aladdin Biochemical Technology Co., Ltd. (Shanghai, China). Cy3 and Rhodamine 6G (R6G) were used as probe molecules and purchased from Macklin Biochemical Co., Ltd. (Shanghai, China). Polyvinylpyrrolidone (PVP K-30), used as a dispersant, was obtained from the same supplier.

The microfluidic syringe pump (XMSP-C) used for the ADDC process was purchased from Nanjing Ximai Nano Technology Co., Ltd. (Nanjing, China). A UV–Vis spectrophotometer (V-630) for monitoring the absorbance of AgNP suspensions was obtained from JASCO Corporation (Tokyo, Japan). The morphology of the fabricated silver nanostructures was characterized using a cold field emission scanning electron microscope (SU8010, Hitachi, Tokyo, Japan). A microarray spotter for Cy3 deposition was equipped with a digital pneumatic microinjection pump (DMP-200), which was purchased from MicroNano Precision Instruments Co., Ltd. (Wuhan, China). Fluorescence measurements of Cy3 on the silver nanostructures were performed using a microarray scanner (GenePix 4100B, Molecular Devices, San Jose, CA, USA). Raman spectra of R6G were acquired using a Raman spectrometer (LabRAM HR Evolution, HORIBA, Kyoto, Japan).

The software used in this study included ImageJ (version ImageJ 1.54g) for binarization of SEM images of the silver nanostructured surface and calculation of AgNPs coverage, and Origin (OriginPro 2024 (64-bit) SR1 10.1.0.178) for all data statistical analysis and graphing.

### 2.2. Fabrication of Silver Nanostructured Surface via ADDC

During the ADDC process, a liquid film forms on the glass substrate and undergoes evaporation-driven self-assembly, where the evaporation behavior directly influences the uniformity of silver nanostructures on the surface. Previous studies have shown that solvents with low surface tension facilitate uniform and controllable self-assembly of nanostructures [45,46]. Therefore, absolute ethanol with a relatively low surface tension of 22.39 mN/m was selected as the solvent. Silver nanoparticle powder with an average diameter of approximately 20 nm was dispersed into ethanol and ultrasonicated for 15 min to obtain a uniformly dispersed AgNP suspension with a concentration of 10 mg/mL.

Polyvinylpyrrolidone (PVP) was added as a stabilizer to maintain the dispersion stability of the AgNP suspension and ensure uniform nanostructure formation during the ADDC process. AgNPs were dispersed in ethanol containing different concentrations of PVP to prepare suspensions with a fixed concentration of 10 mg/mL. The absorbance of the suspensions was monitored for 60 min using a UV–Vis spectrophotometer to evaluate dispersion state and colloidal stability. A relatively stable absorbance indicates good dispersion with minimal aggregation or sedimentation, whereas a decrease in absorbance suggests particle aggregation or settling.

**Figure 1:**
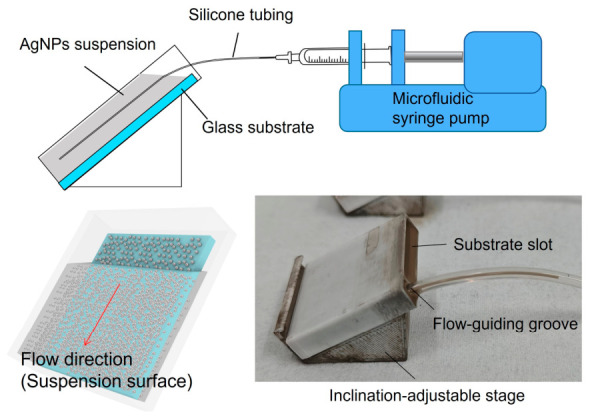
Schematic of the ADDC Experimental Setup.

After obtaining a stable AgNP suspension, silver nanostructured surfaces were fabricated using the ADDC process. As illustrated in Figure 1, the experimental setup mainly consists of a substrate reservoir, an inclination stage, a microfluidic syringe pump, and a sealed fluidic connection system. The substrate reservoir is mounted on the inclination stage, which controls the angle between the substrate and the liquid surface. A side channel is designed in the reservoir, with its bottom connected to a silicone tube for continuous withdrawal of the suspension. The tube is further connected to the syringe pump, forming a closed fluidic system that effectively prevents bubble introduction and liquid leakage during operation. Based on the reservoir design, all substrates have a size of approximately 2.5 cm × 2.5 cm. The prepared AgNP-ethanol suspension was first introduced into the reservoir, and a cleaned glass substrate was placed along the sidewall to ensure positional stability during coating. The suspension was then continuously withdrawn at a preset flow rate. As the liquid level gradually receded, AgNPs were enriched at the liquid film-substrate interface and progressively self-assembled. After complete withdrawal of the suspension, the substrate was removed and dried under ambient conditions, yielding a uniform silver nanostructured surface upon solvent evaporation.

### 2.3. Effect of ADDC Process Parameters on the Morphology of Silver Nanostructured Surface

**Figure 2:**
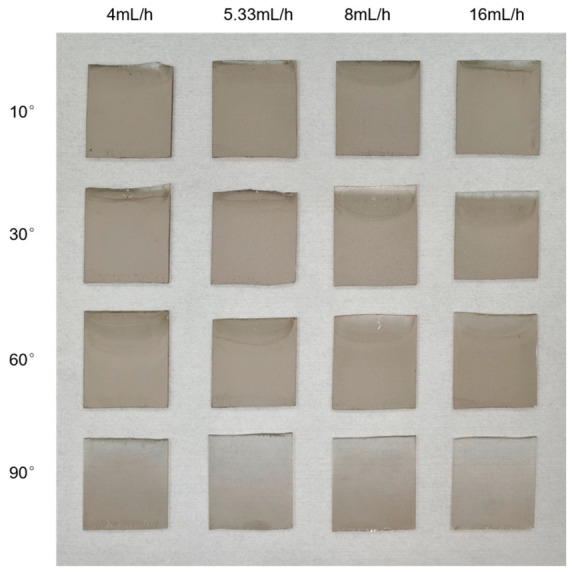
Silver nanostructured surfaces fabricated via various substrate tilt angles and withdrawal flow rates.

During the ADDC process, substrate inclination angle and withdrawal flow rate are the key process parameters that control the formation of silver nanostructures. To systematically evaluate the effects of these parameters on the surface morphology of silver nanostructured surfaces and their fluorescence enhancement performance, a series of silver nanostructured surfaces was prepared using different parameter combinations in this study. The microscopic morphological characteristics and the variation patterns of fluorescence enhancement response with respect to the process parameters were analyzed in detail. Specifically, four substrate inclination angles were selected: 10°, 30°, 60°, and 90°. For each inclination angle, four withdrawal flow rates were applied: 4, 5.33, 8, and 16 mL/h, respectively. This design yielded a total of 16 silver nanostructured surfaces fabricated under distinct processing conditions, as shown in Figure 2. Subsequently, SEM was employed to characterize the surface morphology of the silver nanostructures on each substrate. The uniformity of silver nanoparticle distribution, interparticle gap characteristics, and their systematic variation with processing parameters were examined. To quantitatively assess the packing density of Ag nanoparticles on the substrate surface, SEM images were processed using ImageJ software (version ImageJ 1.54g), and the surface coverage fraction was calculated.

Furthermore, to evaluate the effect of silver nanostructure morphology on the fluorescence enhancement performance of the substrates, Cy3 was employed as a fluorescent probe molecule to systematically compare the fluorescence enhancement capability of the prepared substrates. A 10 nM Cy3 solution was deposited onto each substrate in a 3 × 3 array using a microarray spotter (MicroNano Precision Instruments Co., Ltd., DMP-200, Wuhan, China). After natural drying of the droplets, the Cy3 fluorescence signals on the substrate surfaces were acquired using a microarray fluorescence scanner, and the variations in fluorescence intensity under different preparation conditions were comparatively analyzed.

### 2.4. Evaluation of Fluorescence and SERS Detection Performance of Silver Nanostructured Surface

To systematically evaluate the fluorescence enhancement performance and practical sensing capability of silver nanostructured surfaces fabricated via the ADDC process, Cy3 and R6G were selected as the fluorescent and Raman probe molecules, respectively. These analytes were deposited onto silver nanostructured surfaces prepared under the optimized processing conditions for performance characterization. For fluorescence measurements, Cy3 solutions with concentrations of $10^{-3}$, $10^{-2}$, $10^{-1}$, 1, 10, and $10^{2}$ nM were prepared and deposited onto each substrate surface in a 3 × 3 array format using a microarray spotter. After natural drying under ambient conditions, the fluorescence signals were acquired using a microarray scanner (GenePix 4100B, Molecular Devices, San Jose, CA, USA). The detection limit of Cy3 on the optimized substrate was determined by analyzing the concentration-dependent fluorescence responses. A bare glass slide was used as a control under identical conditions, and the fluorescence enhancement factor was calculated by comparing the fluorescence intensities obtained from the two substrates. For SERS measurements, R6G solutions with concentrations ranging from $10^{-6}$ to $10^{-10}$ M was prepared. A 2 μL aliquot of each solution was deposited onto the optimized silver nanostructured surface. After natural drying of the droplets, the Raman spectra were collected and recorded using a Raman spectrometer (LabRAM HR Evolution, HORIBA, Kyoto, Japan). The detection limit for R6G was determined based on the concentration-dependent Raman responses. In addition, linear regression analysis was performed to evaluate the correlation between Raman signal intensity and analyte concentration.

## 3. Results

### 3.1. Dispersion and Stability Analysis of AgNPs Suspensions

**Figure 3:**
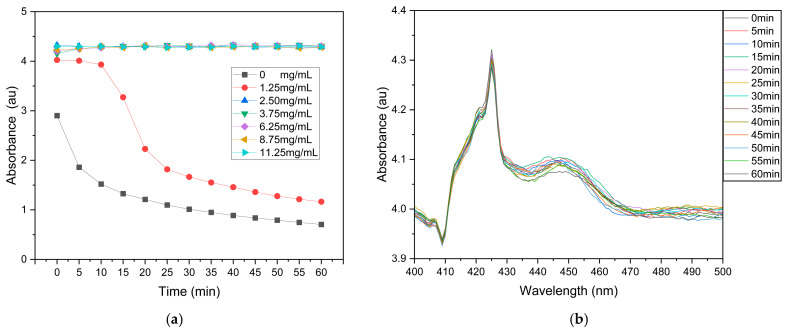
UV-Vis absorbance characterization of the dispersion stability of AgNPs suspensions with PVP: (**a**) variation in absorbance at the LSPR peak (~425 nm) of AgNPs suspensions with different PVP concentrations; (**b**) UV-Vis spectra of AgNPs suspension with a PVP concentration of 2.5 mg/mL over 0–60 min.

To ensure the dispersion stability of AgNPs suspensions during the ADDC process, PVP was introduced as a stabilizing agent. AgNPs suspensions with different PVP concentrations were prepared and thoroughly mixed by ultrasonication, followed by real-time monitoring of their absorption spectra over 0–60 min using a UV–Vis spectrophotometer to evaluate the effect of PVP concentration on suspension stability. Figure 3a presents the time-dependent variation in absorbance at the LSPR peak around 425 nm under different PVP concentrations. The results show that when the PVP concentration is ≥2.5 mg/mL, the absorbance at 425 nm remains nearly unchanged over 60 min, indicating that PVP significantly enhances the dispersion stability of the AgNPs suspension. This effect is mainly attributed to the steric hindrance and electrostatic repulsion provided by the long-chain polymer structure of PVP adsorbed on the nanoparticle surface, which effectively suppresses particle aggregation and sedimentation [47,48]. Furthermore, the full-spectrum absorption of the AgNPs suspension with a PVP concentration of 2.5 mg/mL was analyzed over 0–60 min after ultrasonication, as shown in Figure 3b. No noticeable changes in spectral profile or peak position were observed within 60 min, further confirming the excellent dispersion stability of the system. Based on these results, an AgNPs-ethanol suspension containing 2.5 mg/mL PVP was selected as the optimal formulation to ensure dispersion stability during dip coating and to achieve a uniform silver nanostructured surface via the ADDC process. This formulation was used for all subsequent experiments.

### 3.2. Morphology Evolution of Silver Nanostructured Surfaces and Influence on Fluorescence Enhancement

**Figure 4:**
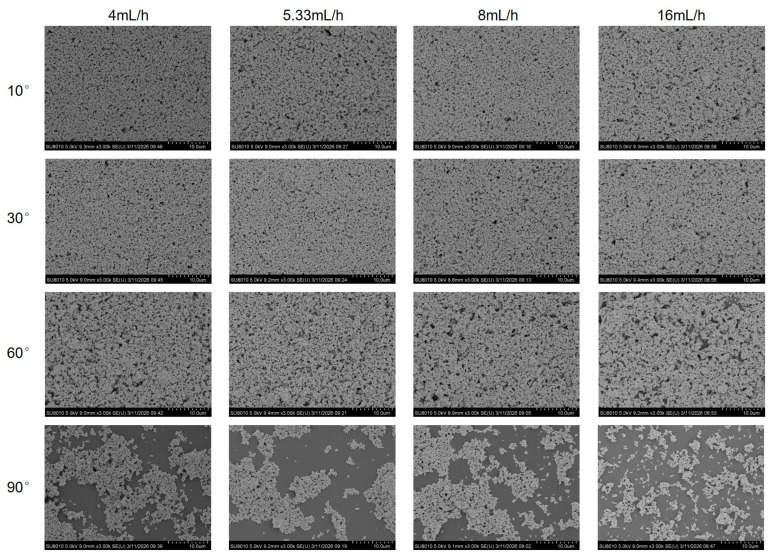
SEM images of silver nanostructured surfaces fabricated under different substrate inclination angles and withdrawal flow rates.

**Figure 5:**
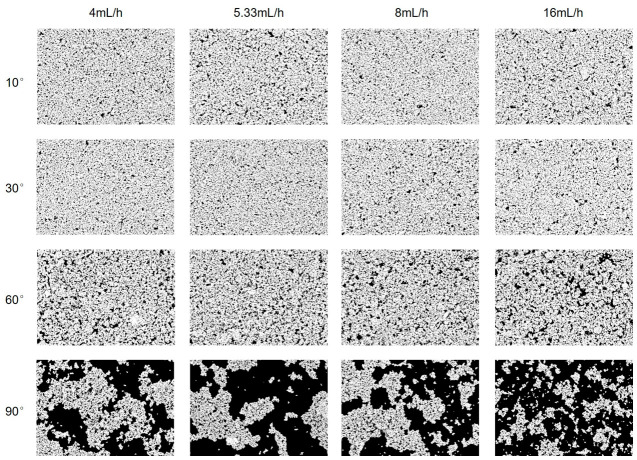
ImageJ binarization results of SEM images of silver nanostructured surfaces under different substrate inclination angles and withdrawal flow rates.

**Figure 6:**
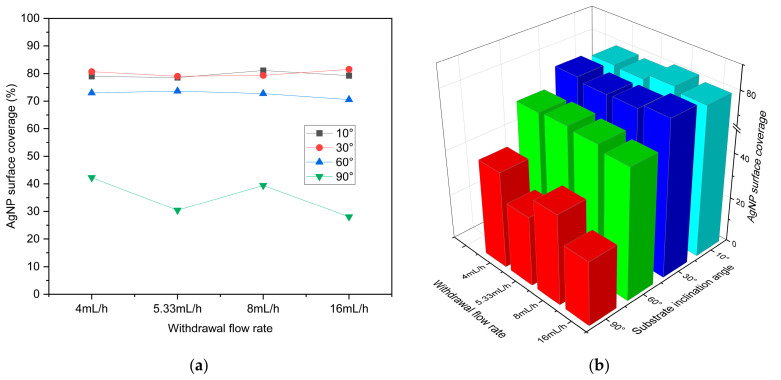
Line plots and 3D bar charts of AgNP surface coverage for silver nanostructured surfaces under different substrate inclination angles and withdrawal flow rates, calculated based on ImageJ analysis. (**a**) Line plots; (**b**) 3D bar chart.

To systematically evaluate the influence of substrate inclination angle and withdrawal flow rate on the morphology of silver nanostructures, a series of silver nanostructured surfaces was fabricated under substrate inclination angles ranging from 10° to 90° and withdrawal flow rates ranging from 4 to 16 mL/h. The corresponding surface morphologies were characterized using SEM, as shown in Figure 4. On this basis, the SEM images were further processed using ImageJ for binarization analysis to quantitatively evaluate the surface coverage of AgNPs. In the binary images, the white regions represent AgNP-covered areas, whereas the black regions correspond to exposed glass slide surfaces, as shown in Figure 5. The statistical results of AgNP surface coverage are presented in Figure 6. The results indicate that, under the same substrate inclination angle, variations in withdrawal flow rate result in only minor fluctuations in AgNP surface coverage, with no significant changes observed in surface morphology, suggesting that withdrawal flow rate has a relatively limited influence on the morphology of silver nanostructured surfaces. In contrast, substrate inclination angle exhibits a much more pronounced effect on AgNP surface coverage and morphological evolution. Specifically, the surface coverage of AgNPs decreases significantly with increasing substrate inclination angle. At inclination angles of 10° and 30°, the AgNP coverage remains relatively high and comparable, with average coverage values of 79.5% and 80.2%, respectively. When the inclination angle increases to 60°, the average coverage decreases to 72.5%, and further declines to 35.1% at 90°. Corresponding SEM images and ImageJ analysis reveal that at inclination angles of 10° and 30°, AgNPs are densely distributed with relatively small exposed regions on the glass slide surface, forming a relatively uniform and continuous silver nanolayer structure. As the inclination angle increases to 60°, the particle distribution density gradually decreases, and the exposed glass slide regions evolve from isolated small-scale areas into partially interconnected structures, while the surface still maintains a certain degree of continuity. When the inclination angle further increases to 90°, AgNPs predominantly exist as discrete clusters, making it difficult to form a continuous coverage layer and resulting in a significant increase in the exposed glass slide area. These results indicate that substrate inclination angle plays a dominant role in governing the morphological evolution of silver nanostructured surfaces.

**Figure 7:**
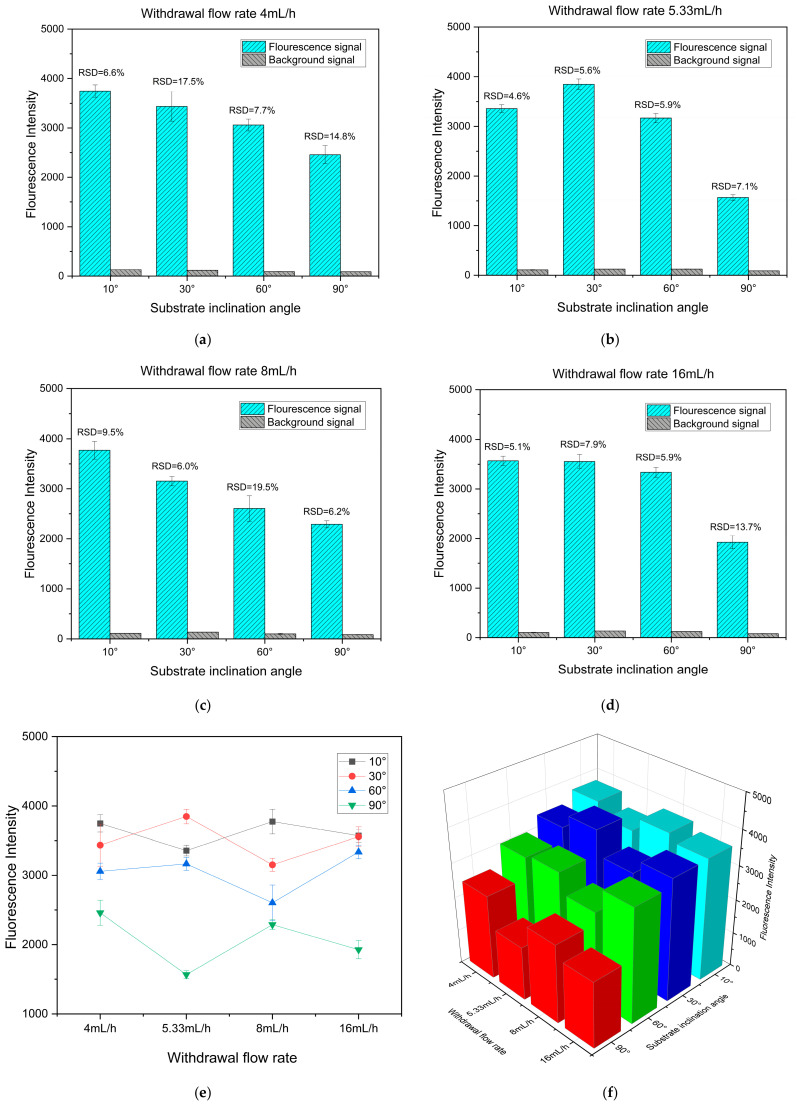
Average Cy3 fluorescence intensity of silver nanostructured surfaces under different substrate inclination angles and withdrawal flow rates (**a**–**d**), along with the corresponding line plot (**e**) and three-dimensional bar chart (**f**).

To further investigate the effects of substrate inclination angle and withdrawal flow rate on the fluorescence enhancement performance of the fabricated silver nanostructured surfaces, a 10 nM Cy3 solution was deposited onto substrates prepared under various inclination angles and withdrawal flow rates. The fluorescence signal intensity of Cy3 on each substrate was then recorded using a microarray scanner, and the results are presented in Figure 7a–d. Meanwhile, the average fluorescence intensity of Cy3 for all samples was statistically analyzed, and corresponding line plots and three-dimensional bar charts were constructed to provide a more intuitive comparison of fluorescence enhancement performance under different parameter conditions, as shown in Figure 7d,e. The results indicate that, under the same substrate inclination angle, the average fluorescence intensity of Cy3 on silver nanostructured surfaces prepared at different withdrawal flow rates shows no significant variation. In contrast, under a fixed withdrawal flow rate, the fluorescence intensity varies markedly with substrate inclination angle, exhibiting an overall increasing trend as the inclination angle decreases. When the inclination angle is reduced to 30° and 10°, the differences in fluorescence intensity gradually diminish. Combined with the evolution of surface morphology with substrate inclination angle, it can be inferred that the fluorescence enhancement performance is closely correlated with the surface structure. As the inclination angle decreases, the distribution of AgNPs on the substrate becomes increasingly dense, which facilitates the formation of more nanoscale gaps and enhances localized electromagnetic field coupling. When the inclination angle is further reduced to 30° and 10°, the differences in surface morphology become less pronounced, and the density of regions with enhanced localized electromagnetic fields tends to stabilize. To verify the reproducibility of the observed trend, substrates were re-fabricated at substrate inclination angles ranging from 30° to 90° under different withdrawal flow rates, followed by fluorescence measurements. The results are presented in Figure S1 and show a consistent variation trend with the aforementioned findings. As shown in the statistical results in Figure 7, although the highest fluorescence intensity was obtained at a substrate inclination angle of 30° and a withdrawal flow rate of 5.33 mL/h, no clear trend in fluorescence intensity with varying withdrawal flow rate was observed within this angle range. Moreover, the fluorescence intensity remained at a relatively high level under all tested withdrawal flow rates, indicating that the withdrawal flow rate has a limited effect on the fluorescence enhancement performance at this inclination angle. Notably, the substrate prepared at a withdrawal flow rate of 16 mL/h still exhibited strong and stable fluorescence enhancement performance, and a higher withdrawal flow rate is beneficial for reducing preparation time and improving fabrication efficiency. Therefore, a substrate inclination angle of 30° and a withdrawal flow rate of 16 mL/h were selected as the preparation conditions for subsequent experiments.

Furthermore, to evaluate the uniformity of the fluorescence signals on different substrates, the relative standard deviation (RSD) was further calculated based on the average fluorescence intensity and corresponding standard deviation obtained from nine independent measurement positions on each substrate. The RSD, defined as the ratio of the standard deviation to the mean value, was used to characterize the dispersion of fluorescence signals, with lower values indicating better signal uniformity. As shown in Figure 7 and Figure S1, both the standard deviations and RSD values of all substrates remain at relatively low levels, demonstrating the excellent uniformity of the fabricated silver nanostructured surfaces. In addition, repeated experiments performed using different fabrication batches exhibited consistent RSD variation trends, indicating good stability and reproducibility of the fluorescence enhancement performance under identical fabrication conditions. These results further confirm the capability of the proposed method for fabricating stable and uniform fluorescence enhancement substrates.

### 3.3. Fluorescence Detection Performance of Silver Nanostructured Surface

**Figure 8:**
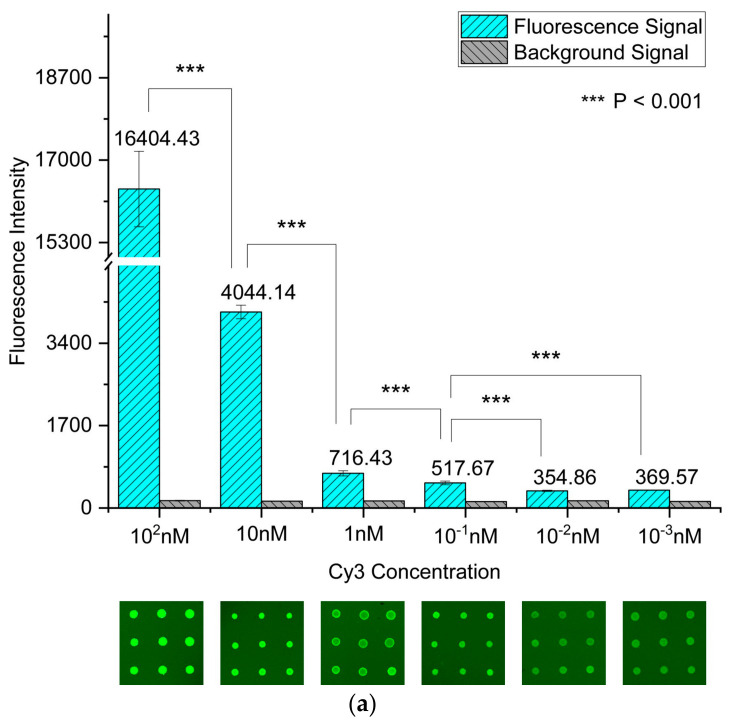

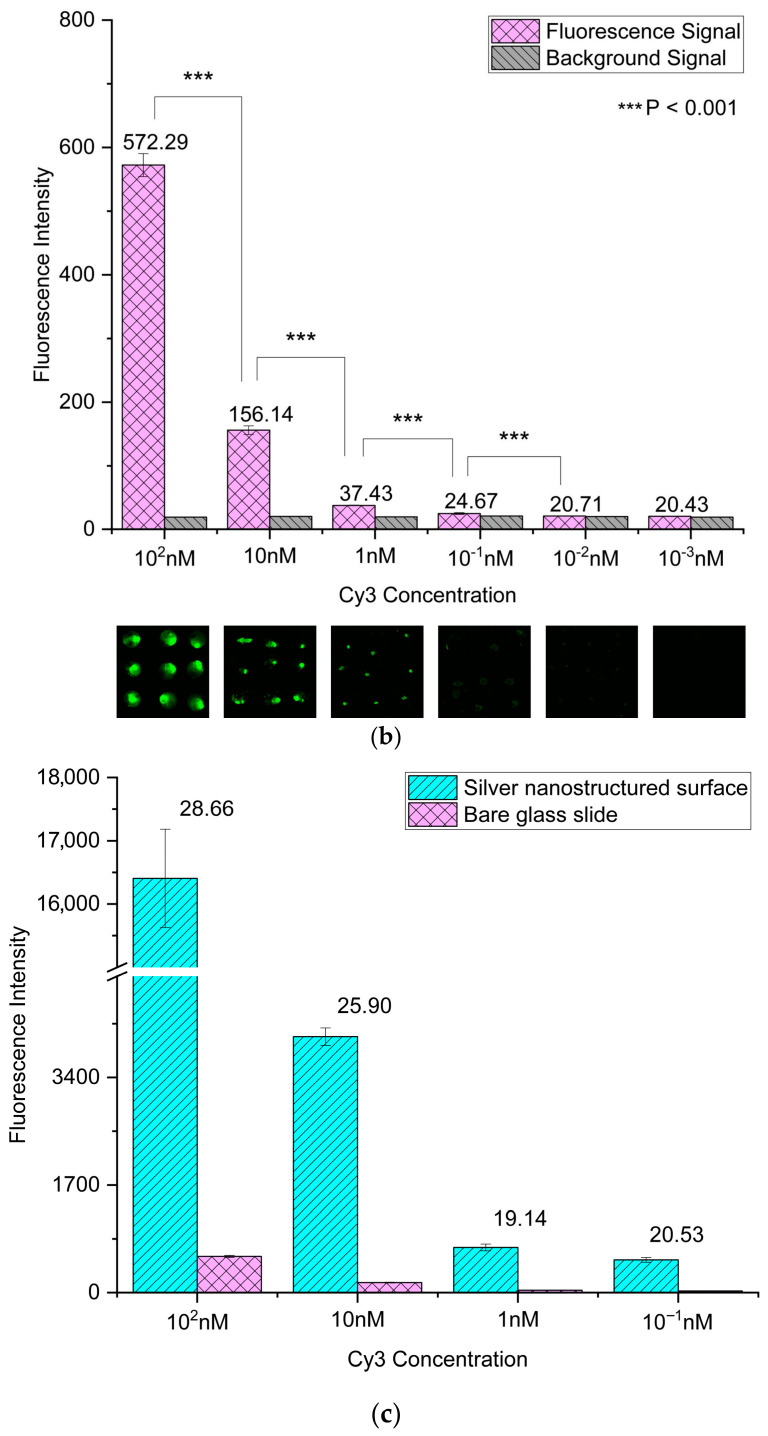
(**a**,**b**) Average fluorescence intensities of Cy3 at different concentrations on silver nanostructured surfaces and a glass slide; (**c**) fluorescence enhancement factor of the silver nanostructured surfaces relative to the glass slide.

To systematically evaluate the fluorescence enhancement performance and practical detection capability of silver nanostructured surfaces fabricated via the ADDC process as MEF substrates, Cy3 was selected as a fluorescent probe molecule. The limit of detection (LOD) of Cy3 on the silver nanostructured surfaces prepared via ADDC was compared with that on a bare glass slide. Furthermore, the fluorescence enhancement factor (FEF) was calculated at different concentrations. The average fluorescence intensities of Cy3 at various concentrations on the two substrates were measured and recorded using a microarray scanner, as shown in Figure 8a,b. For the silver nanostructured surfaces, when the Cy3 concentration was below${\;10}^{-2}$ nM, no statistically significant difference in fluorescence intensity was observed among the low-concentration samples (*p* = 0.19). In contrast, when the concentration increased to${\;10}^{-1}$ nM, a significant difference was observed compared to ${\;10}^{-2}$ nM (*p* < 0.001). Therefore, the LOD of Cy3 on the silver nanostructured surfaces can be determined as${\;10}^{-1}$ nM. By comparison, for the bare glass slide, although statistically significant differences could still be observed at concentrations below 1 nM, the average fluorescence signal was nearly indistinguishable from the background, making reliable detection difficult. Accordingly, the LOD of Cy3 on the glass slide was estimated to be approximately 1 nM. These results demonstrate that the silver nanostructured surfaces fabricated via ADDC improve the fluorescence detection limit of Cy3 by approximately one order of magnitude compared to the bare glass substrate. Furthermore, the fluorescence enhancement factor of the silver nanostructured surfaces relative to the glass slide at Cy3 concentrations ≥ 1 nM was calculated, as shown in Figure 8c. The results indicate that the silver nanostructured surfaces provide a fluorescence enhancement of approximately 19.14–28.66-fold, highlighting their significant advantage in amplifying fluorescence signals and their potential for highly sensitive detection of trace biochemical biomarkers.

### 3.4. SERS Detection Performance of Silver Nanostructured Surface

**Figure 9:**
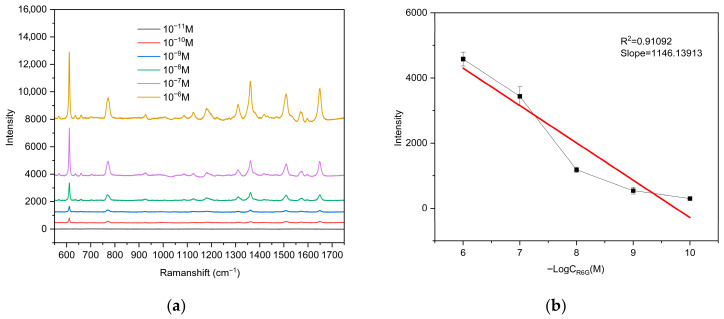
(**a**) Raman spectra of R6G at different concentrations; (**b**) variation in the characteristic peak intensity at 612 cm^−1^ with R6G concentration and the corresponding linear fit; the red line represents the fitting result, with error bars representing the deviation from three measurements.

To systematically evaluate the Raman enhancement performance and practical detection capability of the silver nanostructured surface fabricated via the ADDC process as an SERS substrate, R6G was selected as the probe molecule. The LOD of R6G on the silver nanostructured surface prepared via ADDC was determined, and the enhancement factor (EF) was further calculated. Raman spectra of R6G at various concentrations on the silver nanostructured surface were recorded using a Raman spectrometer (excitation wavelength 532 nm, integration time 1 s, laser power 5 mW, spectral range 550–1750 cm^−1^), as shown in Figure 9a. Characteristic peaks of R6G at 612, 770, 1314, 1364, 1510, and 1650 cm^−1^ can be clearly observed when the concentration is ≥$10^{-10}$. In contrast, when the concentration decreases to${\;10}^{-11}$ M, these characteristic peaks become indistinguishable. Therefore, the LOD of R6G on the ADDC-fabricated silver nanostructured surface is determined to be ${\;10}^{-10}$ M. Furthermore, Raman signals of R6G at different concentrations were collected from three distinct locations on the silver nanostructured surface and averaged. The intensity of the characteristic peak at 612 cm^−1^ was selected as the analytical signal for plotting and linear fitting. The results demonstrate a good linear relationship between the peak intensity at 612 cm^−1^ and the R6G concentration, with a correlation coefficient of *R*^2^ = 0.91092.

Furthermore, the enhancement performance of the fabricated substrate was quantitatively evaluated using the surface-enhanced Raman scattering (SERS) enhancement factor (EF) [49]. The EF is used to characterize the Raman signal amplification capability of a SERS substrate, representing the ratio of the signal intensity generated by a single molecule under SERS conditions to that under non-enhanced Raman conditions. It is defined as follows:

(1) $$EF=\frac{I_{SERS}\times C_{NR}}{I_{NR}\times C_{SERS}}$$

where *I*_SERS_ and *I*_NR_ denote the Raman intensities of the characteristic peak measured on the silver nanostructured surface and the silicon wafer, respectively, while *C*_SERS_ and *C*_NR_ correspond to the concentrations of R6G under the respective conditions. In this study, the characteristic peak intensity of 10^−10^ M R6G at 612 cm^−1^ measured on the silver nanostructured surface (*I*_SERS_ = 341.02) and that of 10^−2^ M R6G on the silicon wafer under the same conditions (*I*_NR_ = 83.85) were substituted into the above equation, yielding an enhancement factor of EF = 4.07 × 10^8^. In summary, the silver nanostructured surface fabricated via the ADDC process exhibits remarkable Raman enhancement capability and high detection sensitivity, providing strong support for the construction of high-performance SERS substrates and trace molecular detection.

### 3.5. Comparative Evaluation of Silver Nanostructured Surface

To facilitate a comparative evaluation of the fluorescence enhancement performance, SERS performance, and application potential of the silver nanostructured surface fabricated via the ADD process proposed in this study, the fabrication strategy and corresponding enhancement performance were systematically compared with representative studies summarized in Table 1 and Table 2.

In terms of fluorescence enhancement performance, for example, Sui N et al. constructed Ag@SiO_2_-DNA-Cy3 nanostructured sensors, and Kaur V et al. designed Ag-coated gold nanostar dimer nanoantennas, achieving Cy3 fluorescence enhancement factors of approximately 2.5-fold and an average of 15-fold, respectively. In addition, substrates fabricated via top-down nanofabrication approaches also exhibit excellent fluorescence enhancement performance. For instance, Yoo H W et al. prepared silver nanodot arrays using nanoimprint lithography, and Xun Lu et al. fabricated silver nanorod substrates via glancing angle deposition, achieving fluorescence enhancement factors of approximately 15.8-fold and 71-fold, respectively. In comparison, the silver nanostructured surfaces developed in this work achieved an average fluorescence enhancement of approximately 19.14–28.66-fold, demonstrating enhancement performance comparable to the aforementioned studies. Meanwhile, the present study enables rapid construction of silver nanostructures through a relatively simplified ADDC process, while maintaining favorable fluorescence enhancement performance and offering improved fabrication simplicity.

In terms of SERS substrate fabrication and sensing performance, for example, Liu L et al. developed Ag@Au core–shell nanoparticle systems via physical deposition and chemical self-assembly, while Wang L et al. prepared Fe_3_O_4_@SiO_2_@Ag magnetic composite particles through a multi-step synthesis process. Both systems exhibited excellent SERS performance and high sensitivity, providing important references for the design of high-performance SERS substrates. In addition, SERS substrates fabricated using top-down nanofabrication approaches, such as anodic aluminum oxide nanoporous structures combined with gold nanorods developed by Fang Z et al. and three-dimensional metal-dielectric-metal structures fabricated by Yue Niu et al., achieved even higher detection sensitivity. In comparison, the silver nanostructured surfaces developed in this work also demonstrated good R6G sensing performance, with a limit of detection of 10^−10^ M and an enhancement factor of 4.07 × 10^8^. Furthermore, the ADDC process offers advantages in terms of fabrication simplicity and efficiency while maintaining favorable sensing performance. Compared with top-down fabrication approaches that rely on multiple complex procedures or high-vacuum systems, the present method enables rapid construction of large-area silver nanostructures through simple dip coating combined with substrate inclination control. The fabrication time is approximately 15 min, and the resulting silver nanostructured surfaces exhibit favorable plasmonic enhancement performance while significantly shortening the preparation cycle.

## 4. Conclusions

The ADDC process, with its simple setup, high efficiency, and tunable nanoscale gaps, offers a convenient and effective approach for fabricating large-area, uniform silver nanostructured surfaces. However, the controllable fabrication of plasmonic substrates for MEF and SERS, as well as the underlying mechanisms by which key process parameters influence structural morphology and optical performance, remain to be systematically elucidated. In this study, a bifunctional silver nanostructured surface with both MEF and SERS capabilities was fabricated based on the ADDC technique. The effects of key process parameters, including substrate inclination angle and withdrawal flow rate, on the morphology and optical properties of the silver nanostructures were systematically investigated. Cy3 and R6G were employed as probe molecules to evaluate the MEF and SERS performance of the silver nanostructured surface prepared under optimal conditions. The results demonstrate that, compared with withdrawal flow rate, the substrate inclination angle exerts a more pronounced influence on surface morphology and fluorescence enhancement performance. As the inclination angle decreases, the self-assembled structure of AgNPs becomes increasingly dense, leading to a significant enhancement in fluorescence performance. When the inclination angle is reduced to 30°, the fabricated substrate exhibits optimal fluorescence enhancement performance. The silver nanostructured surface prepared at a substrate inclination angle of 30° and a withdrawal flow rate of 16 mL/h was further evaluated for its practical detection capability. For fluorescence detection, the substrate enables reliable detection of Cy3 down to $10^{-1}$ nM, representing an improvement of approximately one order of magnitude compared with a bare glass slide, and exhibits a fluorescence enhancement factor of approximately 19.14–28.66-fold over a range of concentrations. For SERS detection, the substrate achieves a detection limit of $10^{-10}$ M for R6G, with an enhancement factor of 4.07 × $10^{8}$ relative to a bare silicon substrate. These results demonstrate that the silver nanostructured surface fabricated via the ADDC process exhibits excellent optical response in both MEF and SERS applications and shows great potential for trace-level molecular detection. Moreover, this work provides an efficient and practical strategy for the controllable fabrication of LSPR-based functional substrates and their nanostructured surfaces. Future studies may be extended to complex biological fluids and environmental sample systems to systematically evaluate the applicability, stability, and anti-interference performance of the substrate under practical detection conditions.

## Figures and Tables

**Table 1:** Comparison of fabrication strategies and performance of different Cy3 fluorescence enhancement substrates or nanomaterials.

	Substrate/Material Classification	Fabrication Strategy and Duration	Cy3 Fluorescence Enhancement Performance
Sui N et al. [50]	Ag@SiO_2_-DNA-Cy3 nanostructured sensor	Ag nanoparticle coating with SiO_2_, 12 h	Cy3 fluorescence intensity increased by approximately 2.5-fold
Kaur V et al. [51]	Ag-coated gold nanostar (Au@Ag NSs) dimer nanoantenna	DNA origami technique; dimerization of rectangular DNA origami monomers, 12 h	Fluorescence enhancement of a single Cy3 molecule up to 65-fold, with an average enhancement of approximately 15-fold
Yoo H W et al. [52]	Silver nanodot array pattern	Platinum-assisted nanoimprint lithography, ~2 h	Approximately 15.8-fold fluorescence enhancement for Cy3-labeled DNA
Xun Lu et al. [53]	Silver nanorod substrate	Glancing angle deposition technique, ~1 h	Fluorescence enhancement of Cy3-labeled MPIF-1 standard antigen increased by approximately 71-fold
This study	Silver nanostructured surface	ADDC, 15 min	Average Cy3 fluorescence enhancement of approximately 19.14–28.66-fold

**Table 2:** Comparison of fabrication strategies and performance of different SERS substrates.

	Substrate/Material Classification	Fabrication Strategy and Duration	LOD of R6G	EF
Liu L et al. [54]	Ag@Au core–shell nanoparticles	Physical deposition and chemical self-assembly, 6 h	10^−10^ M	2 × 10^7^
Wang L et al. [55]	Fe_3_O_4_@SiO_2_@Ag (FSA) magnetic particles	Solvothermal reaction + improved Stöber method + chemical reduction deposition + seed-mediated growth, ~13 h	10^−7^–10^−8^ M	1.34 × 10^5^
Fang Z et al. [56]	Au nanorods (AuNRs) + porous anodic alumina (AAO) nanoporous film	Anodic oxidation for AAO nanopores, 45 min	10^−12^ M	1.02 × 10^9^
Yue Niu et al. [57]	Metal-dielectric-metal (MDM) three-dimensional micropillar array structure	Colloidal lithography, atomic layer deposition, and ion-beam sputtering, ~6 h	10^−12^ M	5.72 × 10^7^
This study	Silver nanostructured surface	ADDC, 15 min	10^−10^ M	4.07 × 10^8^

## Data Availability

The original contributions presented in this study are included in the article. Further inquiries can be directed to the corresponding author.

## Supplementary Materials

The following supporting information can be downloaded at: https://www.mdpi.com/article/10.3390/bios16050292/s1, Figure S1. Average Cy3 fluorescence intensity of silver nanostructured surfaces under different substrate inclination angles and withdrawal flow rates (a–d), along with the corresponding line plot (e) and three-dimensional bar chart (f) (Group 2).

## Abbreviations

The following abbreviations are used in this manuscript:

LSPR	Localized surface plasmon resonance
MEF	Metal-enhanced fluorescence
SERS	Surface-enhanced Raman scattering
ADDC	Angle-dependent dip coating
LB film	Langmuir-Blodget film
AgNP	Silver nanoparticle
R6G	Rhodamine 6G
PVP	Polyvinylpyrrolidone
LOD	Limit of detection
FEF	Fluorescence enhancement factor
EF	Enhancement factor
