# Supplementary material for: Angle-Dependent Dip Coating Strategy for Silver Nanostructured Surface Fabrication with Enhanced Fluorescence and Surface-Enhanced Raman Scattering Properties
Source: Biosensors (Basel). 2026 May 16;16(5):292. doi: 10.3390/bios16050292 (PMC13204508; doi:10.3390/bios16050292)
Supplement: Supplementary file 1 [file biosensors-16-00292-s001.zip › biosensors-4282849-supplementary.pdf]

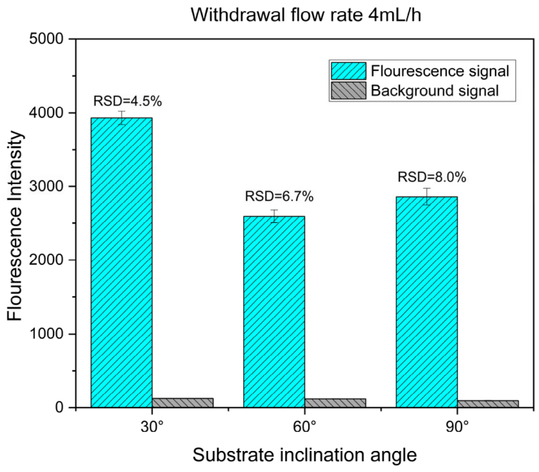

(**a**)

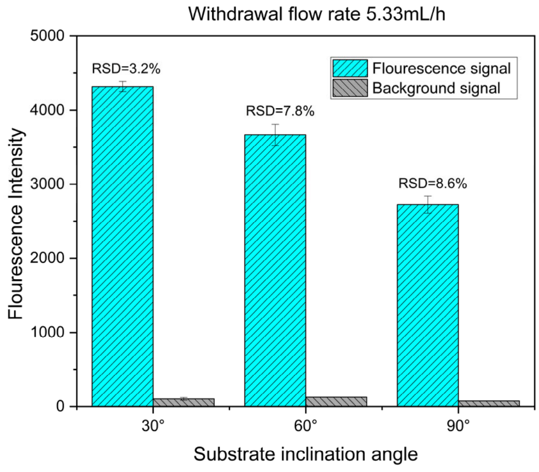

(**b**)

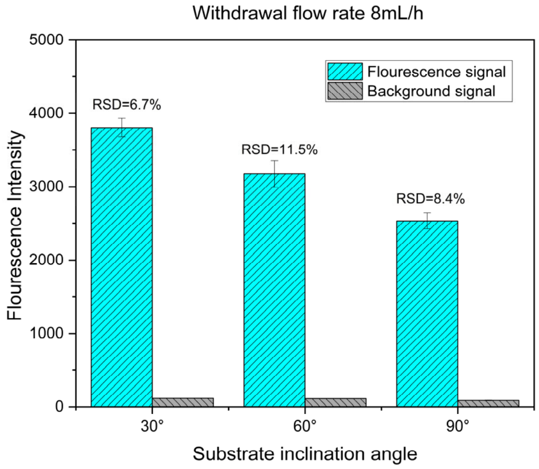

(**c**)

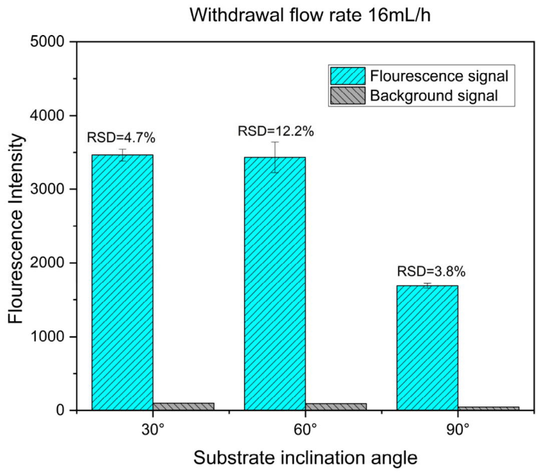

(**d**)

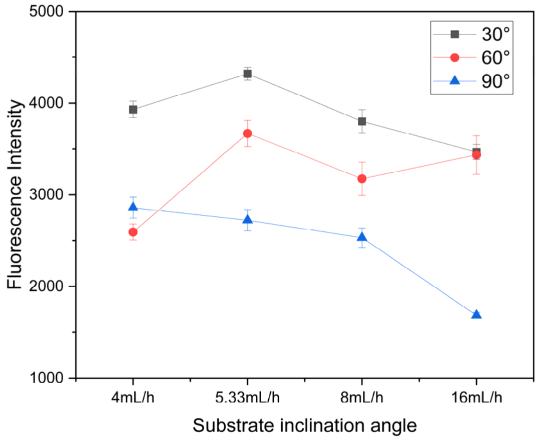

(**e**)

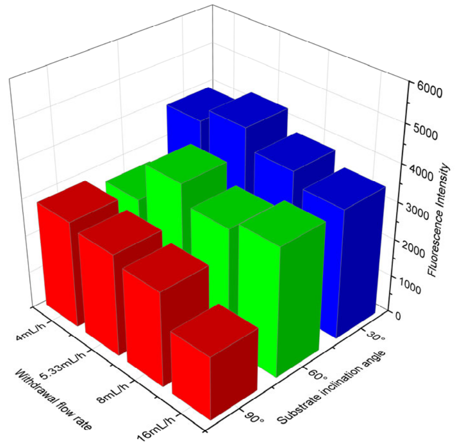

(**f**)

Figure S1. Average Cy3 fluorescence intensity of silver nanostructured surfaces under different substrate inclination angles and withdrawal flow rates (a–d), along with the corresponding line plot (e) and three-dimensional bar chart (f) (Group 2).
